# Knowledge, attitudes, and practices of registered dietitians and nutritionists regarding enteral and parenteral nutrition support in Ghana: a needs assessment study

**DOI:** 10.3389/fnut.2023.1197610

**Published:** 2023-06-29

**Authors:** Ruthfirst E. A. Ayande, Percival D. Agordoh, Vanessa J. Salino, Clarisa Webster-Ariyan, Lindsay Collier, Matilda Asante, Elena T. Carbone

**Affiliations:** ^1^Department of Food and Nutrition, Yale-New Haven Hospital, New Haven, CT, United States; ^2^Department of Nutrition, University of Massachusetts Amherst, Amherst, MA, United States; ^3^Department of Nutrition and Dietetics, University of Health and Allied Sciences, Ho, Ghana; ^4^Department of Dietetics, School of Biomedical and Allied Health Sciences, College of Health Sciences, University of Ghana, Accra, Ghana

**Keywords:** enteral nutrition, parenteral nutrition, nutrition support, dietitian, nutrition professionals, Ghana

## Abstract

**Background:**

There is a paucity of data on enteral and parenteral (EN and PN) nutrition support (NS) provided by nutrition and dietetic practitioners in adult acute care settings in Ghana. Furthermore, gray literature suggests that Ghanaian clinical nutrition professionals (CNPs) are seldom involved in advanced nutrition care teams.

**Objectives:**

To assess the knowledge, attitudes, and practices of Ghanaian CNPs regarding EN and PN.

**Methods:**

An online cross-sectional survey was administered to Ghanaian CNPs ahead of a professional development workshop on EN and PN Support. Participants were asked questions about initiation and timing of NS, and knowledge on availability of commercial formula. A 5-point Likert scale was used to assess self-efficacy in using EN and PN. To assess practical knowledge on EN and PN, participants were asked to identify whether EN or PN was indicated for seven short case scenarios. Open-ended questions were used to assess reasons for participant self-ratings.

**Results:**

A total of 76 dietitians, nutritionists, students, and interns completed the survey. For EN, self-efficacy scores were lowest for the calculation of enteral goal rate, and goal volume (mean 3.20 ± 1.27), and writing of EN prescriptions (mean 3.07 ± 1.29). Self-efficacy scores for the formulation of alternative formulas in lieu of commercial formula were the highest (3.63 ± 1.36). For PN, self-efficacy scores for all domains were lower than 3, with the lowest scores observed for writing PN prescriptions (2.19 ± 1.14) and determining micronutrient additives (2.12 ± 1.04). We identified limited training and lack of practical exposure to NS, limited ability to effectively monitor tube feeds, and prohibitive cost and limited availability of EN and PN formula among the barriers impacting self-efficacy scores.

**Conclusion:**

Given the vital role that CNPs play in the delivery of EN and PN, it is important to develop professional training programs especially focused on PN to bridge knowledge and practice gaps.

## 1. Background

Both enteral and parenteral nutrition support have been used to deliver nutrients to patients in acute care settings to reduce the risk of malnutrition, improve disease prognosis, and reduce hospital length of stay (LOS), among other outcomes (1, 2). Given the highly specialized nature of enteral and parenteral nutrition support (sometimes referred to by the authors as advanced nutrition support), associations like the American Society for Parenteral and Enteral Nutrition (ASPEN) and the European Society for Clinical Nutrition and Metabolism (ESPEN) provide resources to nutrition professionals who are interested in pursuing careers in nutrition support. These include professional development products like webinars and podcasts, and resources and links to accredited professional certifications in nutrition support (3, 4). While their certifications are internationally recognized and available to dietitians outside of the US and Europe, the costs might be prohibitive to some nutrition professionals working in sub-Saharan Africa.

ASPEN and ESPEN periodically provide up-to-date guidelines on nutrition support which are useful references that provide tools for the safe initiation and use of enteral and parenteral nutrition (1, 5, 6). In Ghana and the West African region at large, there are no known societies or organizations focused on advanced nutrition support (7). Additionally, knowledge, attitudes, and practices of nutrition professionals regarding enteral and parenteral nutrition support has not been extensively studied.

Ghanaian clinical nutrition professionals (CNPs) have continued to rely on locally available solutions to mitigate the risk of malnutrition, especially in the pediatric population. With the help of governmental and non-governmental agencies, they have delivered several oral nutritional supplementation programs, including the community-based management of acute malnutrition (CMAM) program (8) to help mitigate malnutrition both at the clinical and community levels. These have helped improve maternal and child nutritional status throughout the country (9, 10). In severely malnourished hospitalized children for example, the CMAM protocol recommends use of the therapeutic milk-based formula F-75 as the initial line of therapy to stabilize patients while they receive treatment for any identified medical complications (usually 2–7 days). Provision of F-100 after the stabilization phase is then made to help the children with their catch-up growth (11). Due to cost and limited availability of these commercial formulas, they are usually prepared in-house by nutrition professionals from reconstituted milk powder, vegetable oil, and sugar with addition of a vitamin and mineral mix. Another product that is integral to the CMAM program is Ready-to-use Therapeutic Food (RUTF), which is an energy and nutrient dense peanut-based supplement provided to children who have malnutrition and are 6 months and older. This is also used among vulnerable adults with severe malnutrition including pregnant and lactating women, people with HIV and/or tuberculosis, and geriatric populations (12).

Although there are extensive guidelines for the treatment of acute severe malnutrition in children and some adults through the CMAM program (9), there is limited information on nutrition support, specifically enteral and parenteral nutrition support, in critically ill adult patients. For adult patients in critical condition and unable to eat by mouth, it is unclear from the literature if Ghanaian nutrition professionals are directly involved in the provision of tube feeding recommendations and how much expertise they have, to make such recommendations. The limited research studies available on nutrition support in Ghana also suggest that there are limited to no guidelines used within health care facilities to provide nutrition support to critically ill patients, so that individual practitioners mainly adopt published ASPEN/ESPEN guidelines to make their own personal protocols (13, 14). Additionally, it appears that nutrition professionals are not always part of advanced nutrition care teams. This has the potential to impact their knowledge base on nutrition support from limited exposure.

Based on the current scope of practice around the world, dietitians are an integral part of the nutrition support team and are the health care professionals required to make prescriptions and provide tube feeding recommendations for patients in need of nutrition support. Given the socio-economic disadvantage of some patients in Ghana and the limited resources available for medical nutrition therapy, it is possible that dietitians currently equipped with the skills to deliver these services may face challenges, including access to commercial formulas, in providing advanced nutrition support. Unfortunately, the enteral and parenteral nutrition experiences and knowledge base of practicing CNPs in Ghana is poorly understood. Research is therefore needed to identify existing knowledge gaps among nutrition professionals so that appropriate programming and training can be developed to help bridge these gaps as well as to develop standard guidelines for the provision of advanced nutrition support in the Ghanaian context. Therefore, this study aimed to assess the knowledge gaps and barriers to providing advanced nutrition support to patients in clinical care settings in Ghana.

## 2. Methods

### 2.1. Study design and participants

A cross-sectional needs assessment survey was administered to Ghanaian dietitians, nutritionists, nutrition and dietetic students, and dietetic interns. Dietitians and Nutritionists are considered two separate designations in Ghana, with separate requirements for credentialling. While dietitians usually work in the clinical setting, nutritionists tend to work in the public health/community setting. Nevertheless, there are some overlaps in the roles that they play. For instance, some nutritionists who work with the CMAM program receive in-service training/specialization to enable them to provide tube feedings. To that end, it was important to include nutritionists in our assessments. Two weeks ahead of a planned remote professional development workshop on Advanced Nutrition Support, participants were invited to complete an online survey to identify their knowledge, attitudes, and practices (KAPs) regarding enteral and parenteral nutrition support. This also served as a pre-test for the planned workshops. We opted for an online survey because the advent of the COVID-19 pandemic made online meetings popular and feasible among nutrition and dietetic professionals in Ghana.

No sample size estimates were calculated for this project. According to the leadership of the Ghana Academy of Nutrition and Dietetics (GAND), the total number of dietitians and nutritionists registered with the Academy is about 400. Approximately twice this number (800) are students/interns (undergraduates and graduate students at various stages of their training), yet less than a quarter of these students were registered with GAND at the time of the study (personal communication with Mr. Agordoh, Vice President of GAND). Of the total number of registered members, the number who usually participate in continuous professional development (CPD) workshops is 100 to 150. Given the fact that there are so few practicing CNPs in Ghana, we aimed to reach all nutrition professionals who historically worked with the Academy through its continuing professional development workshops. Therefore, being a currently active dietitian, nutritionist, nutrition/dietetics student, or dietetic intern, and willingness to complete the survey were the only criteria for participation. The study was reviewed and deemed exempt by the institutional review board at the University of Massachusetts Amherst, and all participants signed an informed consent form online before completing the survey. Links to the online survey were shared using the official WhatsApp page of the Ghana Academy of Nutrition and Dietetics, with a script describing details of the study. Once participants completed the consent forms, they were directed to the main survey. Daily reminders were posted on the WhatsApp page, and participants who had incomplete surveys received daily reminders via emails sent through REDCap.

### 2.2. Research instruments

The online surveys were self-administered using REDCap (see Supplemental data). Of note, this needs assessment was part of a broader project, and participants completed 2 surveys, one prior to a planned workshop and the other after the workshop. The needs assessment survey comprised a total of 31 questions: 6 related to nutrition support practices and types of formula used in participants’ facilities; 12 related enteral nutrition (EN) knowledge and EN self-efficacy; and 13 related to parenteral nutrition (PN) knowledge and PN self-efficacy. Participants were asked questions about their knowledge regarding indications for enteral and parenteral nutrition, timing of nutrition support, and commercial supplements available on the Ghanaian market.

To reduce participant burden, simplify IRB application for exempt review, and ensure utmost privacy and confidentiality of participants, demographic information was not collected from participants. Email addresses and hospital affiliation were the only identifiable information collected (some hospitals may have only one or two dietitians, making hospital affiliation identifiable information). Participants were asked to indicate whether they were a dietitian, nutritionist, or student/intern, as well as provide their number of years of work practice where applicable. The data was de-identified during the analysis phase of the project.

### 2.3. Evaluation of enteral and parenteral nutrition support practices in the clinical nutrition setting

All participants were asked to indicate the name of the hospital or facility in Ghana where they worked in an open-ended question. They were also asked using a binary “yes,” “no” question if their facilities provided clinical nutrition support services to inpatients. To evaluate current enteral and parenteral nutrition support practices in facilities that offered nutrition support, respondents were asked to answer questions regarding the proportion of patients who received nutritional support based on ASPEN/ESPEN guidelines, types of malnutrition screening tools typically used to assess patients, types of professionals who usually performed the screening, and types of enteral and parenteral nutrition formula typically used in their facility, if any.

### 2.4. Assessment of self-efficacy in using enteral and parenteral nutrition

A 5-point Likert scale was used to assess participants’ self-efficacy using enteral and parenteral nutrition support. We were interested in 5 domains of self-efficacy for each aspect of nutrition support. Domains for EN were (1) indications for EN, (2) selection of EN Formula, (3) calculation of goal volume (GV) and goal rate (GR) of EN; (4) writing of EN prescriptions and (5) preparation of alternative enteral formula in lieu of commercial formula. Domains for PN were (1) indications for TPN; (2) calculation of macronutrient provision of PN; (3) calculation of GV and GR of TPN infusion; (4) writing TPN prescriptions; (5) determining TPN micronutrient additives. Enteral and parenteral nutrition had 5 questions each, and each question was scored 1 through 5 (1 = lowest, 5 = highest). Therefore, the highest possible score after summing all 5 questions was 25 for enteral and 25 for parenteral nutrition.

To assess overall confidence in prescribing enteral and parenteral nutrition, participants were asked to rate themselves on a scale of 1 to10 (1 = least confident, 10 = most confident) in terms of their general comfort level using enteral and parenteral nutrition support. To assess factors related to self-efficacy scores, we included 2 open-ended questions, a single question repeated for enteral nutrition and the other for parenteral nutrition, asking participants to explain the reasons for allocating their scores.

### 2.5. Assessment of practical knowledge on the indications for enteral and parenteral nutrition

To assess practical knowledge about enteral and parenteral nutrition support, participants were asked to identify whether enteral or parenteral nutrition support was indicated for seven short case scenarios. The case scenarios were: (1) paralytic gastric ileus, (2) poor PO intake in hemodialysis patient, (3) poor PO intake in patient with pancreatic cancer, (4) small bowel obstruction (5) partial small bowel obstruction with gastrostomy-jejunostomy tube with venting gastrostomy port, (6) malnourished patient with BMI of 13 kg/m^2^, intubated, unable to obtain access for feeds, (7) patient with gastroparesis with residuals consistently 450–600 ml. One point was assigned for each correct answer, and no point for an incorrect answer (1, 0 respectively) making a total of seven possible points for the practical knowledge aspect of the survey. The case scenarios were based on study questions provided to Yale-New Haven Hospital dietetic interns during their medical ICU rotations (Please see questionnaire from Supplemental data).

### 2.6. Assessment of student and intern exposure to enteral and parenteral nutrition support

Participants who identified themselves as students or dietetic interns were asked to answer 8 student-specific questions related to level and field of study, and prior exposure to enteral and parenteral nutrition in class or on rotation. An open-ended question was used to collect responses on what their idea of enteral and parenteral nutrition support entailed either from class, personal studies, or rotations.

Students were asked to complete the practical knowledge sections of the survey but were not required to complete the self-efficacy portions of the questionnaires.

### 2.7. Statistical analyses

Statistical analyses were conducted using IBM SPSS Stats Version 28.0 for Windows (IBM Corporation). Chi-square analyses were conducted for qualitative variables to test for associations between professional background and years of experience, and knowledge of enteral and parenteral formula available on the Ghanaian market. A *p* value ≤0.05 was considered statistically significant. Open ended questions were analyzed qualitatively, by first manually coding the data and then thematizing responses to identify emergent themes.

## 3. Results and discussion

A total of 76 dietitians, nutritionists, students, and interns completed the needs assessment survey (Table 1). The number of initial survey responses to the consent forms was 111. Out of these, there were 100 completed consent forms and 11 incomplete consent forms (forms that were not included if they had not been signed by participants). By the closing time of the pre-workshop surveys, there were a total of 77 completed pre-workshop surveys (out of the 100 completed consent forms). Out of these, 7 were duplicates (cross-referenced with participant email addresses). These were excluded, leaving the total number of completed surveys at 70. Upon further quality control checks, 6 surveys were identified as surveys that could count as competed surveys—likely were not read into the system as complete due to glitches. These were manually coded as complete and included to the 70 completed surveys, bringing the total number of completed pre-test surveys to 76. Please see Supplemental data (Supplementary Figure 1) for participant flowsheet.

**Table 1:** Occupational characteristics of participants.

	*N* (%)
Professional background (all participants)	
Nutritionist	12 (16)
Registered dietitian	47 (62)
Student/intern	17 (22)
Total	76 (100%)
Years of experience for nutrition professionals	
Years	*N* (%)
3 or less	20 (34)
4 or more	39 (66)
Total	59 (100)

Most participants self-identified as registered dietitians (62%), followed by Nutritionists (16%), and students or interns (22%). Among dietitians and Nutritionists, two thirds (66%) reported 4 or more years of work experience (Table 1). The clinical settings in which the dietitians and nutritionists worked (32 hospitals) included teaching hospitals, district and mission hospitals, regional/military/police hospitals, private hospitals, and a quaternary hospital. These hospitals together were situated in 10 out of the 16 regions of the country (Table 2).

**Table 2:** Classification of hospitals where respondents work.

Classification	Number of Hospitals	Regions where Hospitals are located
Teaching Hospitals	6	Greater Accra, Ashanti, Central, Volta, Northern
District/Mission Hospitals	10	Greater Accra, Ashanti, Central, Eastern, Western, Bono, Ahafo, Northern, North-Eastern
Regional/Military/Police Hospitals	8	Greater Accra, Northern, Bono
Private Hospitals	7	Greater Accra, Northern, Ashanti
Quaternary Hospital	1	Greater Accra
Total	32 hospitals	10 regions total*

* Participant hospitals were located in 10 out of 16 regions in Ghana, representing 63% of the regions.

### 3.1. Nutrition screening practices for acutely ill patients

Most participants (*n* = 62, 82%) indicated that their facility provided clinical nutrition services to inpatients. Of the participants who reported providing clinical nutrition services to hospitalized patients 65% (*n* = 40), indicated that nutrition screening was routinely practiced, while 35% reported either no routine screening or did not know. According to ASPEN guidelines, nutrition risk screening is recommended for all hospitalized patients in the ICU to identify those who might benefit the most from enteral nutrition (15). Providing standard guidelines for the Ghanaian context could help increase the number of facilities that perform routine nutrition screening as part of patient care.

**Figure 1:**
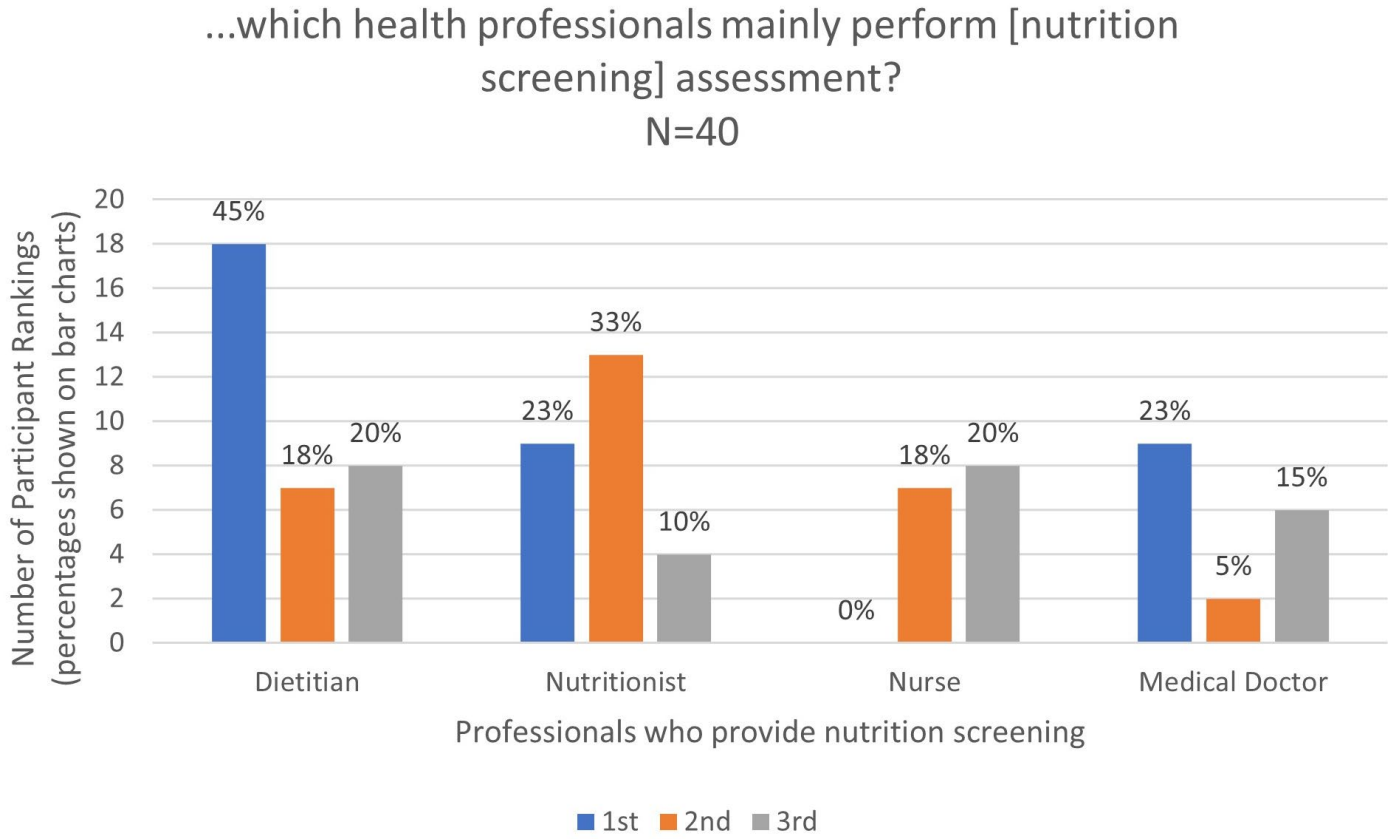
Ranking of professionals who perform nutrition screening.

Based on the responses provided by the 40 participants who selected “yes” for nutrition screening, dietitians were ranked first by most of the participants (45%) as the main professionals performing nutrition screening in their facility, followed by Nutritionists, who were ranked second by 33% of participants (Figure 1). In a UK-based study assessing nutrition support attitudes and knowledge among health care providers, there was strong agreement that dietitians were responsible for decisions regarding nutrition support, although in their study there was also strong agreement that anesthetist/intensivists and medical and surgical teams were responsible for decision making, demonstrating that it was unclear to participants who was responsible for nutrition support decision-making (16). None of the respondents in our study ranked nurses first. Indeed, the same number of participants (*N* = 9, 23%) ranked medical doctors and nutritionists as the main professionals who conducted nutritional screening of patients. While this was unexpected given the central role that nurses play in patient care in Ghana and deviates from the UK study where nurses were considered integral to nutrition support decision making (16), this finding is consistent with results from a Europe-wide survey that showed minimal involvement of nurses in performing nutritional assessments in ICU settings (17). In developing nutrition support teams, it would be useful for dietitians, nutritionists, and nurses to work together to provide screening protocols for critically ill patients.

### 3.2. Nutrition screening tools

Most participants (90%) indicated that BMI or body weight assessments (including one response for Bioelectric impedance analysis) were the main screening methods used in their facilities. While BMI is a convenient, easy-to measure screening tool, it may not be reflective of nutritional status when used on its own. It is the current position of the [American] Academy of Nutrition and Dietetics that the Malnutrition Screening Tool (MST) be used to screen adults for malnutrition regardless of their age, medical history, or setting (18). However, only 33% of participants who performed screening in their facilities indicated that the MST was used. Given that the MST is also an easy to administer tool and asks questions about weight loss (and degree of weight loss), and appetite or food intake, considering it in protocols for Ghanaian dietitians might be useful.

### 3.3. Initiation of nutrition support

Less than half of participants who provided inpatient clinical services (*n* = 24, 39%) indicated that patients were usually kept for <48 h prior to initiation of nutrition support. However, 21% reported that they did not know or were not sure. This elucidates the challenge identified from literature that CNPs are not always part of nutrition support teams in Ghana. Additionally, combined, the number of participants indicating that patients are not screened within 48 h was 40%. This percentage suggests the need for providing protocols for nutrition screening upon admission to the ICU to promote early initiation of nutrition support.

For patients with indications for enteral nutrition, 28% of respondents indicated that <10% of them did receive enteral nutrition within 48 h. Again, many of the participants (25%) did not know or were not sure, which is expected given our results regarding initiation of nutrition support.

### 3.4. Types of EN and PN formula used in facilities

Majority of participants (63%) indicated that kitchen-prepared blenderized tube feeds (BTF) were the main formula used for enteral nutrition in their facilities, followed by commercial ready-to-use formula (50%). Future protocols for enteral nutrition in Ghana must take this into consideration and possibly provide guidance for the use of BTF.

When asked to list the types of formula used for tube feeds, the majority of the participants who listed commercial formula listed oral nutrition supplements rather than enteral formula (brand names withheld). While the oral nutrition supplements that were mentioned were most comparable to 1.0 and 1.5 kcaL/ml polymeric formula from the same company and could be used in the short term for tube feeds, it is not ideal for use as sole source nutrition for a prolonged period. This is because it is more likely to exceed micronutrient RDAs while using it to meet daily Caloric goals. For instance, based on the nutritional information provided on its label, a single cartoon of the 1.5 kcaL/ml oral nutrition supplement mentioned by participants is designed to meet 25–50% of the RDAs for most micronutrients. Most oral nutrition supplements also have a higher amount of sugar, making them hypertonic, compared to enteral formula. This might enhance tube feed blockage and degradation if used for a prolonged period (19). There is limited literature about what the typical ICU LOS is in Ghana. In a study published among burns patients in the Komfo Anokye teaching hospital, the mean LOS for each year, over a 4-year analysis, ranged between 6 and 9 days (20). Among patients with surgical site infections in the Korle Bu teaching hospital, post-op hospitalization ranged between a day for limb amputations to 16 days for rectal surgeries (21). Among COVID patients, ICU stays ranged up to 7 days (22), and based on field observations, ICU stays could generally range between 10 and 21 days (personal communication with Mr. Agordoh, Vice President of GAND). For short-term use, the benefits of oral nutrition supplement as tube feeds might outweigh the risk of overnutrition. However, unless otherwise contraindicated, polymeric isotonic enteral formula are recommended as the gold standard for enteral nutrition for patients in the ICU (15). We recommend that future studies assess ICU LOS and other clinical outcomes of patients on different types of oral and enteral nutrition formula in Ghana.

About a third of participants in our study (37.1%) indicated that no PN formula was used in their facilities, while some of the participants did not know which PN formulas were used in their facilities (30.6%). Two-in-one and three-in-one solutions were indicated by 10 and 9% of participants, respectively, suggesting the limited access to PN formula in Ghana. Two participants (3.2%) who selected the “other” option on the survey mentioned that they used single nutrient solutions including dextrose, amino acid, and fat emulsions (brand names withheld). Given the limited knowledge of facility-specific PN formula among many respondents, it is important that health facilities provide in-service training on formulary as part of dietitian orientation so that they are aware of formulas available in their institutions.

### 3.5. Knowledge of enteral and parenteral nutrition formula on the Ghanaian market

About half (51%) of the participants reported that they had no knowledge of the enteral or parenteral nutrition formulas available on the Ghanaian market. Chi-square analyses showed no statistical differences in knowledge of available enteral and parenteral nutrition supplements/formulas based on profession (dietitian vs. nutritionist). There were also no statistical differences in knowledge of the available enteral formula based on number of years of practice (Table 3). Similarly, there were no differences in knowledge of parenteral nutrition formula by profession or number of years of practice (Table 3). Given the relatively fewer number of nutritionists who participated in the study, it is likely that there was not enough statistical power to detect true difference between the two groups.

**Table 3:** Knowledge of available enteral and parenteral nutrition formula according to profession and number of years of practice.

	Professional practice				Number of years of practice*			
	Nutritionists *N* = 12	Dietitians *N* = 47	Total *N* = 59	value of *p* (2-sided)	3 years or less *N* = 20	4 years or more *N* = 39	Total *N* = 59	value of *p* (2-sided)
Do you know of any enteral nutrition (EN) formulas available on the Ghanaian market?								
Yes	4 (33%)	25 (53%)	29 (49.2%)	0.333	7 (35%)	22 (56%)	29 (49.2%)	0.170
Do you know of any parenteral nutrition (PN) formulas available on the Ghanaian market?								
Yes	4 (33%)	17 (36%)	21 (36%)	1.000	5 (25%)	16 (41%)	21 (36%)	0.263

* Years of experience dichotomized due to small sample sizes.

### 3.6. Nutrition professionals’ self efficacy and practical knowledge in prescribing and using enteral and parenteral nutrition

Table 4 shows the mean scores for self-efficacy of nutrition professionals regarding the use of enteral and parenteral nutrition support. Scores for each question (maximum score = 5), as well as for the sum of scores for all questions (maximum score = 25) are shown here. Scores for overall confidence level (maximum score = 10) and practical knowledge (maximum score = 7) are also displayed.

**Table 4:** Self-efficacy, knowledge, and practice of enteral and parenteral nutrition support among ghanaian dietitians and nutritionists.

	Mean score (SD)
Enteral nutrition	
Indications for EN	3.51 (1.24)
Selection of EN formula	3.32 (1.25)
Calculation of GV and GR of EN	3.20 (1.27)
Writing EN prescriptions	3.07 (1.29)
Preparation of an alternative enteral formula in lieu of commercial formula	3.63 (1.36)
EN self-efficacy (total)^1^	16.73 (5.84)
Overall confidence in prescribing and using EN (2)	5.27 (3.08)
Parenteral nutrition	
Indications for TPN	2.75 (1.27)
Calculation of macros	2.57 (1.34)
Calculation of GV and GR of TPN	2.34 (1.20)
Writing TPN prescriptions	2.19 (1.14)
Determining TPN micronutrient additives	2.12 (1.05)
PN self-efficacy (total)^1^	11.92 (5.56)
Overall confidence in prescribing and using PN^2^	2.75 (2.56)
Knowledge and Practice Domain	
Practical Knowledge in EN and PN^3^	3.16 (1.92)

EN, enteral nutrition; GV, goal volume; GR, goal rate; PN, parenteral nutrition; macros, macronutrients; TPN, total parenteral nutrition. *N* = 59.

1 Total possible score is 25.

2 Total possible score is 10.

3 Total possible score is 7.

For enteral nutrition, self-efficacy scores were lowest in the calculation of enteral goal rate and goal volume (mean 3.20 ± 1.27) and writing enteral nutrition prescriptions (mean 3.07 ± 1.29). Self-efficacy scores were highest for the formulation of alternative enteral formulas in lieu of commercial formula (3.63 ± 1.36).

For parenteral nutrition, self-efficacy scores for all domains were lower than 3, with the lowest scores observed for writing parenteral nutrition prescriptions (2.19 ± 1.14) and determining micronutrient additives (2.12 ± 1.04).

Consistent with the above results, participants rated themselves lower in overall confidence in their ability to prescribe and use parenteral nutrition (2.75 ± 2.56) compared with enteral nutrition (5.27 ± 3.08). Our results show that there are more deficits in knowledge and practice of parenteral nutrition compared to enteral nutrition. Training protocols focused on parenteral nutrition support are warranted, and in-service specialization might be needed to help bridge this knowledge gap.

### 3.7. Underlying factors related to low self-efficacy

We identified two themes that described underlying factors for low self-efficacy in the use of enteral and parenteral nutrition support among Ghanaian nutrition professionals: limited training and exposure to nutrition support during professional education and practice, and limited knowledge of available formula for administering enteral and parenteral nutrition support. Some participants reported exposure to enteral and/or parenteral nutrition support through online courses, overseas training, and hands on experience in the work setting. However, most reported they had limited training and were rarely exposed to nutrition support in their practice setting. These results are consistent with findings from a similar study performed among health professionals in the UK, who cited lack of knowledge, no clear guidelines, or unclear responsibilities as barriers to delivery of nutrition support (16). In their study, sources of knowledge for dietitians were largely from reading (>50%), followed by additional courses (>40%), and work-based training (30%). Less than 30% of the dietitians in their study cited University training and nutrition teams as sources of knowledge on nutrition support. This shows that even within settings where dietitians are expected to have more experience with nutrition support, continuous professional development post-University training is an integral part of their competency building for nutrition support therapy.

Some participants in our study mentioned that they did not know what types of formula were available on the Ghanaian market. Given the low confidence of many nutrition professionals to administer these important aspects of nutrition care, additional training and specialization could be made available to them to help increase their knowledge regarding delivery of enteral and parenteral nutrition, including exposure to information about types of formula available on the market. More importantly, intensive training to improve skills related to calculation of GV and GR of both EN and PN is required, given that these were the areas where the participants had the lowest self-efficacy. This would make them valuable members of the medical team as they would be able to make recommendations to other health practitioners and patients about both appropriate formula type and tube feed administration.

### 3.8. Challenges with administering enteral and parenteral nutrition

In addition to the themes that appear to characterize reasons for the perceived low confidence in the use of enteral and parenteral nutrition among this group of participants, some participants identified challenges that directly related to the administration of enteral and parenteral nutrition support. Three themes emerged for this (1) monitoring of patients, (2) cost of commercial formula, and (3) availability of commercial formulas. Some participants reported that even when enteral nutrition was available and used in their practice setting, monitoring of feeds was a challenge. While additional information was not provided by participants, a plausible explanation for this finding could be due to lack of skilled personnel. The only published study on nursing protocols for nasogastric feeding in Ghana suggests that there are limited opportunities for in-service training and insufficient tube feeding protocols available to nurses (14). Limited availability of commercial formula was another recurring theme from the open-ended questions. From the responses, many nutrition professionals who did provide enteral nutrition support usually provided blenderized tube feeds (BTF). Although BTF have several advantages, including promotion of a diverse microbiome, better tolerance to enteral feeds, and potential cost savings, there are some challenges, including concerns with safety and nutritional adequacy (23). Additionally, a recent Master’s thesis assessing the nutritional composition of hospital prepared blends (fortified soups), identified that there were no standardized recipes available, and the blends were not nutritionally adequate (24).

Protocols that provide standardized recipes and modular additives to improve macronutrient and micronutrient composition could be warranted given the socioeconomic context of Ghana, where dietitians rely more on BTF than on higher costing commercial formulas for the provision of enteral nutrition. Additionally, given food safety concerns with the use of BTF, protocols should also include guidelines for safe handling of ingredients, bolus vs. continuous feeding, appropriate hang time of feeds, and policy regarding source of feeds (e.g., feeds brought from home vs. hospital prepared feeds). Training modules should also educate on the contraindications for the use of BTFs, and appropriate equipment (for example specifications for French size of tubes) among other things.

### 3.9. Student knowledge of enteral and parenteral nutrition support

Less than 50 % of students in our sample (47%) reported that they had encountered information on nutrition support in their training. Twenty nine percent (29%) of them reported that they had encountered information on parenteral nutrition support in their clinical rotations, whereas 71% of students reported that they encountered information on enteral nutrition support during rotations (Table 5). The sample of students and interns was small (*N* = 17) and is therefore not representative of all dietetic students and interns across the country. However, given the fact that the results demonstrate and mirror the need for training on parenteral nutrition compared to enteral nutrition, advocating for more robust training for students and interns both in the classroom and on clinical rotations is warranted.

**Table 5:** Student and intern exposure to enteral and parenteral nutrition support.

Question	Yes
Have you encountered advanced nutrition support (enteral and parenteral nutrition) in your training yet?	8 (47%)
Have you encountered any enteral nutrition on your rotations?	12 (71%)
Have you encountered any parenteral nutrition on your rotations?	5 (29%)

### 3.10. Strengths and limitations of study

Our study questionnaire (see Supplemental data) contained extensive questions related to nutrition support practices in the clinical setting in Ghana, including nutrition screening practices in hospitals, accessibility of formula, and knowledgebase of practitioners regarding EN and PN. To keep participant burden as minimal as possible, the practice assessment aspect of the questionnaire was limited to participants correctly identifying the indications for EN and PN from seven short case studies. Admittedly, knowledge of the indications for EN and PN on their own might not necessarily reflect practical knowledge of EN and PN. We however believe that this serves as a good proxy to provide some baseline data on this aspect of EN and PN knowledge to aid with future education.

Additionally, while we made efforts to include all CNPs who historically participated in CPDs, 76 participants completed our needs assessment survey, which limited our statistical power to detect differences in knowledge based on profession and years of practice. That said, it is noteworthy that these professionals were affiliated to 32 hospitals across 10 out of the 16 regions of the country. The 63% representation of the country’s regions could be suggestive that the data regarding nutrition support practices might be similar across hospitals. Generalizations of these findings should however be done with caution, and future studies specifically exploring regional differences across all the regions of the country might be beneficial. Regarding the findings for students, generalizability is unknown as we had a very small sample of students.

Finally, our study assessed knowledge, attitudes, and practices of CNPs in Ghana but did not examine clinical outcomes of patients in ICU settings in Ghana. Future studies designed to examine outcomes of patients on nutrition support are needed to complement our findings as efforts continue for identifying areas of improvement for future training.

### 3.11. Implications for research and practice

Our study aimed to identify gaps in enteral and parenteral nutrition knowledge and practices among Ghanaian dietitians, nutritionists, students, and dietetic interns. We found low self-efficacy scores in performing some aspects of enteral nutrition support and even lower scores for parenteral nutrition support. Participants were least confident in their ability to calculate enteral goal rate, goal volume, and writing prescriptions. On the other hand, respondents were most confident in their ability to “formulate” alternative formulas in the absence of commercial enteral nutrition formula. Additionally, participants had low confidence in all domains of PN measured, with the least confidence in parenteral nutrition prescriptions and determining micronutrient additives. We identified one master’s thesis that assessed nutrition support practices in 17 Ghanaian health facilities (13). This project targeted all health professionals and found among other things that there were limited standards in the delivery of nutrition support in the respondent’s facilities (13). While this is grey literature, it provides insights into the paucity of research on the question of nutrition support in Ghana and foregrounds the need for continued research in the area. To our knowledge, our study is the first in Ghana to characterize the extent of the gap and the first to do so exclusively among Ghanaian nutrition professionals.

Given the key role that dietetic and nutrition professionals play in the delivery of EN and PN, it is important to develop professional training programs to bridge gaps in knowledge and practice. Further, based on our results, there is a greater need for parenteral nutrition training compared to enteral nutrition, and this must be considered when formulating training programs. This is useful for programming continuous professional development for currently practicing dietitians and nutritionists, as well as future professionals. Furthermore, given that the student participants reported a lack of exposure to enteral and parenteral nutrition support on their rotations, it is important that specialized rotations in this field are developed and made available to this group of future dietetics professionals who might be interested in pursuing careers in advanced nutrition support. This might require additional training for dietitians already in active practice who may in turn serve as professional mentors and preceptors for students and interns.

## Data availability statement

The data that support the findings of this study are available from the corresponding author upon reasonable request.

## Ethics statement

This study involving human participants was reviewed and approved by the Institutional review board at the University of Massachusetts Amherst. All participants provided their written informed consent to participate in this study.

## Author contributions

RA and PA: conception and design (80% and 20% respectively) and manuscript drafting. RA, PA, EC, and MA: contribution to acquisition, analysis, or interpretation of data. RA, PA, VS, CW-A, LC, MA, and EC: critical revision. All authors contributed to the article and approved the submitted version.

## Funding

RA is a Spaulding-Smith fellow of the University of Massachusetts Amherst graduate school, as well as a 2022/23 recipient of a Margaret McNamara Education Grant (https://www.mmeg.org/).

## Conflict of interest

The authors declare that the research was conducted in the absence of any commercial or financial relationships that could be construed as a potential conflict of interest.

## Publisher’s note

All claims expressed in this article are solely those of the authors and do not necessarily represent those of their affiliated organizations, or those of the publisher, the editors and the reviewers. Any product that may be evaluated in this article, or claim that may be made by its manufacturer, is not guaranteed or endorsed by the publisher.

## Abbreviations

ASPEN: American Society for Parenteral and Enteral Nutrition
CMAM: Community-based Management of Acute Malnutrition
CNPs: clinical nutrition practitioners
CPD: continuous professional development
EN: enteral nutrition
ESPEN: European Society for Clinical Nutrition and Metabolism
GAND: Ghana Academy of Nutrition and Dietetics
GV: Goal volume
GR: Goal rate
ICU: Intensive care unit
KAP: Knowledge, attitudes, practices
LOS: Length of stay
NS: Nutrition support
PN: Parenteral nutrition
PO: per OS (by mouth)
Post-op: Post-operation
RUTF: Ready-to-use therapeutic food
