# Supplementary material for: Knowledge, attitudes, and practices of registered dietitians and nutritionists regarding enteral and parenteral nutrition support in Ghana: a needs assessment study
Source: Front Nutr. 2023 Jun 29;10:1197610. doi: 10.3389/fnut.2023.1197610 (PMC10339799; doi:10.3389/fnut.2023.1197610)
Supplement: Supplementary file 1 [file Data_Sheet_1.PDF]

Advanced NS: Pre-survey and Needs Assessment

Hello! Thank you for taking time to complete the Nutrition Support Survey. The full survey comprises two short questionnaires, a preliminary survey to help us prepare for the advanced nutrition support workshop in July, and a post-completion survey that will be shared later to help us assess the impact of the workshop.

This questionnaire you are about to complete is the preliminary survey, which will collect some brief information about your professional background and also ask you some questions about your experience with enteral and parenteral nutrition support in Ghana.

Please complete the following questions about your professional background and facility-based nutrition support practices

1 Email address

____

First name only (or initials)

____

Please choose which of these best describes your current profession.
Note: If you are registered professional and a student, please select your professional status. Eg: an RD who is currently back in school for graduate studies should select "Registered Dietitian"

- ◯ Nutritionist
- ◯ Registered Dietitian
- ◯ Student/Intern

How many years have you worked as a dietitian or nutritionist?

- ◯ 1 or less
- ◯ 2-3
- ◯ 4-5
- ◯ more than 5

In which hospital or facility do you work in Ghana?

____

In your work setting, do you provide clinical nutrition services to inpatients?

Note: This includes hospitals, CHPS compounds and other facilities that provide clinical services

- ◯ Yes
- ◯ No

Is nutrition screening routinely practiced in your hospital or facility?

- ◯ Yes
- ◯ No
- ◯ Don't Know

If yes, which health professionals mainly perform this assessment?

Note: Use 1, 2, & 3 to rank the top 3 professionals accordingly [1 being the person mostly responsible for performing the assessment]. If only 1 professional performs this in your facilty, select 1 for that professional and move to the next question

Professional

Rank (please rank only 3 professionals)

Dietitian

______

Nutritionist

______

Nurse (General)

______

Nurse (ICU-trained)

______

Medical Doctor (General Practitioner)

______

Medical Doctor (Specialist)

______

Other Professional (please list any other professional)

______

______

Which nutrition screening/assessment methods are used in your facility? Select all that apply	☐ BMI/body weight assessment<br>☐ Subjective Global Assessment (SGA)<br>☐ Malnutrition Universal Screening Tool (MUST)<br>☐ Malnutrition Screening Tool (MST)<br>☐ Mini Nutrition Assessment - Short Form (MNA-SF)<br>☐ Nutrition Risk Index<br>☐ Don't know/not sure<br>☐ Other (please specify below)
Specify other nutrition screening tools used in your facility	__________________________________
In your facility, for how long are patients kept, on average, before any form of nutrition support is initiated?	◯ < 48 hours<br>◯ 2 to 3 days<br>◯ 4 to 7 days<br>◯ >7 days, < 14 days<br>◯ > 14 days<br>◯ Don't know/not sure
What types of EN formula are used in your facility? Note: Select all that apply	☐ None<br>☐ Ready to use (commercial) formula<br>☐ Kitchen-prepared formula from commercial concentrate or powder<br>☐ Kitchen-prepared blenderized tube feeds<br>☐ Don't Know<br>☐ Other (please specify below)
Specify the other types of EN formula used in your facility<br>Note: Please list them in the space and separate with commas	__________________________________

In your opinion, what proportion of patients in your facility with indications for EN, do receive EN within 48 hours?	◯ < 10%<br>◯ 11% to 20%<br>◯ 21% to 30%<br>◯ 31% to 40%<br>◯ >40%<br>◯ Don't know/not sure<br>◯ Other
What types of PN formula are used in your facility?<br>Note: Select all that apply	☐ None<br>☐ 2-in-1 commercial solution<br>☐ 3-in-1 commercial solution<br>☐ Pharmacist prepared formula<br>☐ Don't Know<br>☐ Other (please specify below)
Specify the other types of PN formula used in your facility<br>Note: Please list them in the space and separate with commas	__________________________

Please complete this section to help us assess enteral nutrition knowledge among Ghanaian dietitians and nutritionists

Do you know of any enteral nutrition (EN) formulas available in Ghana for tube feeding?	◯ Yes<br>◯ No
Please list the names of the EN formulas you are aware of on the Ghanaian market.<br>Note: Separate their names with commas	__________________________
On a scale of 1-10, how comfortable are you prescribing enteral nutrition in general?<br>Note: Use the slider bar to indicate your level of comfort; tap and slide till you reach your desired score.	Not comfortable — Somewhat comfortable — Very Comfortable<br>*(Place a mark on the scale above)*
Why did you give yourself this score? Please explain further.<br><br>Note: Eg, if your comfort level with prescribing enteral nutrition is high, tell us why. If it is not that high, tell us why	__________________________

Please choose the option that best describes your knowledge or comfort level regarding certain aspects of enteral nutrition support in the clinical setting

	Never	Almost never	Sometimes	Often	Almost always
1. I am able to assess and determine indications for enteral nutrition support in the clinical setting	◯	◯	◯	◯	◯

2. I am able to determine appropriate enteral nutrition formula type for patients depending on their medical conditions and or metabolic demands	◯	◯	◯	◯	◯
3. I am able to calculate goal volume and goal rate of enteral nutrition formula for a client	◯	◯	◯	◯	◯
4. I am able to write prescriptions for enteral nutrition, including specifying volume advancement	◯	◯	◯	◯	◯
5. I am able to prepare alternative enteral nutrition formulations for my patients when commercial ones are not availabe	◯	◯	◯	◯	◯

Please let us know how much you agree or disagree with these statements regarding the accessibility of enteral nutrition formula on the Ghanaian market, as well as the possible acceptability of Ghanaian patients to enteral nutrition. You don't need to know the exact answer, just give us an idea what you believe to be true.

	Strongly disagree	Disagree	Neither agree nor disagree	Agree	Strongly agree
I believe (or know) that there is a wide variety of enteral nutrition formula available on the Ghanaian market	◯	◯	◯	◯	◯
I believe (or know) that enteral nutrition formula is too expensive for most Ghanaian patients	◯	◯	◯	◯	◯
I believe (or know) that most Ghanaian patients will not accept enteral nutrition	◯	◯	◯	◯	◯

Please complete this section to help us assess parenteral nutrition knowledge among Ghanaian dietitians and nutritionists

Do you know of any parenteral nutrition (PN) formulas available in Ghana?

◯ Yes
◯ No

Please list the names of the PN formulas you are aware of on the Ghanaian market.
Note: Separate their names with commas

____

On a scale of 1-10, how comfortable are you using prescribing parenteral nutrition in general?
Note: Use the slider bar to indicate your level of comfort; tap and slide till you reach your desired score.

Not comfortable — Somewhat comfortable — Very Comfortable

(Place a mark on the scale above)

Why did you give yourself this score? Please explain further.

Note: Eg, if your comfort level with prescribing parenteral nutrition is high, tell us why. If it is not that high, tell us why

____

Please choose the option that best describes your knowledge or comfort level regarding certain aspects of parenteral nutrition support in the clinical setting

	Never	Almost never	Sometimes	Often	Almost always
1. I am able to assess and determine indications for parenteral nutrition support in the clinical setting	○	○	○	○	○
2. I am able to calculate the total calories and macronutrient distribution from dextrose, protein and fat provided by a specific parenteral nutrition prescription or formula	○	○	○	○	○
3. I am able to calculate goal volume and goal rate of parenteral nutrition for a patient based on their estimated needs	○	○	○	○	○
4. I am able to write prescriptions for parenteral nutrition, including dextrose advancement	○	○	○	○	○
5. I am able to determine appropriate micronutrient additives for parenteral nutrition	○	○	○	○	○

Please let us know how much you agree or disagree with these statements regarding the accessibility of parenteral nutrition(PN) products on the Ghanaian market, as well and the role you and other health care workers play in providing PN to patients. You don't need to know the exact answer, just give us an idea what you believe to be true.

	Strongly disagree	Disagree	Neither agree nor disagree	Agree	Strongly agree

1. I know (or believe) that most PN and TPN products in Ghana are preformulated (in other words, they are not formulated at the hospital and are purchased ready to use)	◯	◯	◯	◯	◯
2. I know at least 1 TPN pharmacist in my facility who formulates PN/TPN for patients	◯	◯	◯	◯	◯
3. I know at least 2 preformulated TPN products on the Ghanaian market	◯	◯	◯	◯	◯
4. I am usually an integral part of PN/TPN formulation and prescription in the facility/facilities where I work	◯	◯	◯	◯	◯

For Students and Interns

Level of study	◯ Undergraduate ◯ Graduate (Masters/PhD) ◯ Dietetic Intern
Field of Study	◯ Nutrition ◯ Dietetics
Please select the year you're in on the Program. Note: If you choose "other" please specify	◯ First year<br>◯ Second year<br>◯ Third year<br>◯ Fourth year<br>◯ Other
Specify other year	________________________________
Have you covered advanced nutrition support (enteral nutrition and parenteral nutrition) in your training yet?	◯ Yes<br>◯ No<br>◯ Don't Know
Have you encountered any enteral nutrition (any type of tube feeding) on your rotations?	◯ Yes<br>◯ No<br>◯ Don't Know<br>◯ Not applicable
What do you know about enteral nutrition (either from class, on rotation, or from your own readings)?	____
Have you encountered any parenteral nutrition (peripheral or total PN) on your rotations?	◯ Yes<br>◯ No<br>◯ Don't Know<br>◯ Not applicable
What do you know about parenteral nutrition (either from class, on rotation, or from your own readings)?	____

For the following situations/conditions, indicate whether tube feeds (EN) or parenteral nutrition (PN) would be indicated for the patient if the plan is to feed the patient today. Please answer to the best of your ability.

Condition

Please select if you would use EN or PN

Comments (use if you'd like to explain your answer further)

1. Paralytic gastric ileus

______

______

2. Poor PO intake in hemodialysis patient

______

______

3. Poor PO intake in patient with pancreatic cancer

______

______

4. Small bowel obstruction (SBO)

______

______

5. Partial small bowel obstruction with GJ tube with venting G port

______

______

6. Malnourished patient with BMI 13, intubated, unable to obtain access for feeds

______

______

7. Patient with gastroparesis with residuals consistently 450-600ml

______

______

Optional Section

Do you currently have any enteral nutrition formula in your office or close to you?	◯ Yes<br>◯ No
Please share some pictures if you are able to. To do this, hit the "upload file" bottom and then select the picture(s) you wish to share	
Exclude?	◯ Yes<br>◯ No
Reason for exclusion	◯ Duplicate<br>◯ Testing<br>◯ Other
Specify other reason	__________________________________
Accept as complete? Use for partially complete surveys with significant data.	◯ yes
