# Supplementary figures and images for: Knowledge, attitudes, and practices of registered dietitians and nutritionists regarding enteral and parenteral nutrition support in Ghana: a needs assessment study
Source: Front Nutr. 2023 Jun 29;10:1197610. doi: 10.3389/fnut.2023.1197610 (PMC10339799; doi:10.3389/fnut.2023.1197610)

## Participant Flowchart for needs assessment survey

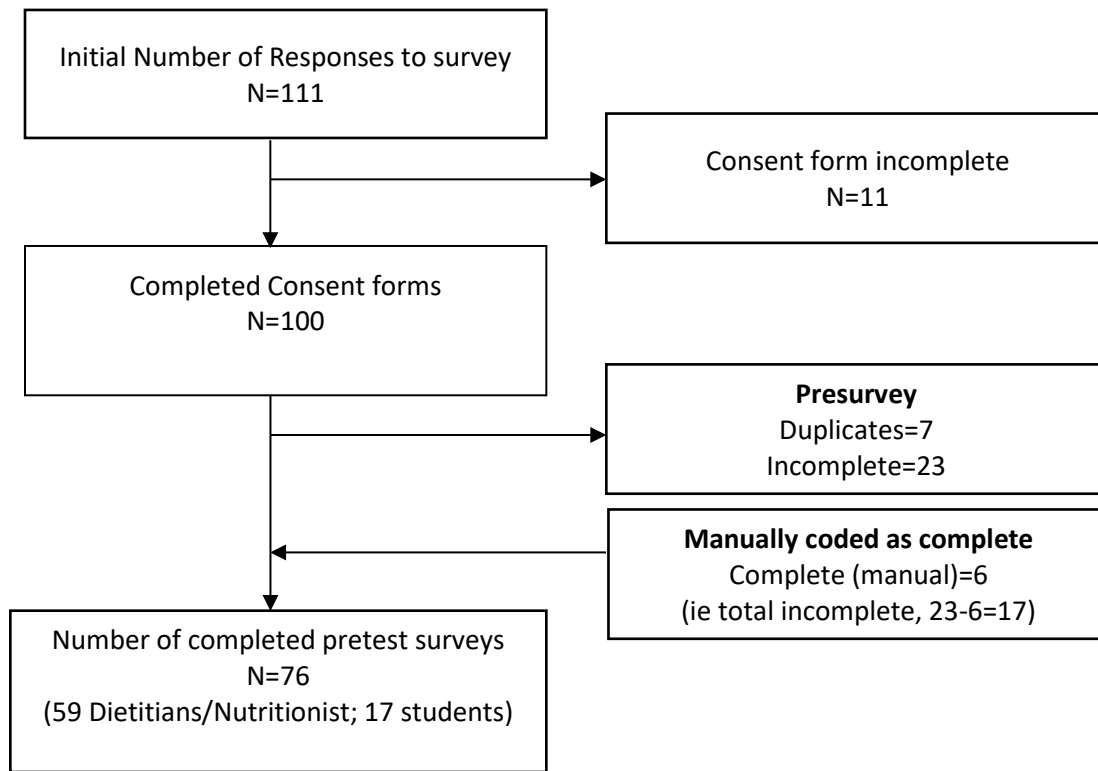

Supplement: Supplementary file 2 [file Image_1.pdf]
